# “I wasn’t scared of the dope I had anymore because I could test it myself:” A qualitative study among rural Appalachians who use drugs about experiences with a fentanyl test strip intervention

**DOI:** 10.21203/rs.3.rs-8139118/v1

**Published:** 2026-02-05

**Authors:** Monica Fadanelli, Tasfia Jahangir, Zora Kesich, Kenneth Lane, April Young, Hannah Cooper

**Affiliations:** Emory University; Emory University; Emory University; University of Kentucky; University of Kentucky; Emory University

**Keywords:** fentanyl test strip, rural, risk environment, overdose

## Abstract

**Background:**

While fentanyl overdose rates are high in rural Appalachia, access to fentanyl test strips (FTS) and related research remains nascent in this region. Here, we explore the perspectives of rural Appalachian people who use drugs (PWUD) about an FTS education and distribution intervention.

**Methods:**

This qualitative study sampled PWUD participants in a harm reduction intervention located in six rural Appalachian Kentucky counties that included FTS education and distribution. During one-on-one semistructured interviews, interviewers queried participants’ perceptions of risk environment features that shape their interest in FTS; their experience of the FTS intervention itself and their use of FTS; and their responses to positive test results. Constructivist thematic analysis methods were applied to transcripts.

**Results:**

PWUD reported that fentanyl saturated local drug markets and that fentanyl overdoses were pervasive and devastating features of these small rural risk environments. Just seven of the 29 participants had heard of FTS before participating in the intervention and only one had a current way to access them. Participants deemed FTS an essential intervention because (1) EMS response to overdoses was unreliable in these rural risk environments where PWUD often used in unmapped areas far from EMS stations; and (2) naloxone reversals, though essential, were physically and emotionally wrenching. Participants found FTS easy to use. Of the 11 participants who reported receiving a positive FTS result, all but one reported engaging in at least one behavior to reduce their overdose risk. Additionally, reflecting social support networks in this risk environment, 1/3 of the 29 participants reported disseminating FTS throughout their networks.

**Conclusions:**

This analysis found that specific features of the local rural Appalachian risk environment render FTS especially important. Unreliable EMS responses enhanced interest in this intervention. Strong social networks, a well-documented strength of rural Appalachian risk environments, permitted unanticipated diffusion of the intervention. Future interventional research should intentionally leverage these networks to extend reach.

## Introduction

In the US, rural Appalachian counties continue to suffer higher rates of fentanyl overdoses and deaths (1). In 2022, overdose mortality rates among adults (aged 25–54) were 64% higher in Appalachia than the national rate (1–3). Extensive research conducted in urban areas has established that fentanyl testing strips (FTS) are an effective overdose prevention tool with high uptake (4–7). FTS are rapid immunoassays originally developed for urine screening that have been repurposed by people who use drugs (PWUD) to detect fentanyl and its analogues in drug samples (7). Using a small amount of residue dissolved in water, the strips provide results within minutes and are highly sensitive (8). Unfortunately, FTS interventions and related research have not followed fentanyl into hard-hit rural areas. This qualitative analysis explores experiences with an FTS education and dissemination intervention among rural Appalachian PWUD.

Multiple studies in urban settings indicate that when PWUD have access to FTS, they utilize the technology at a high rate (9–11). For example, a study with PWUD in New York City found that 77.8% of people who received FTS reported using them to test their substances. Further, this evidence also indicates that PWUD who receive a positive result for fentanyl engage in a range of overdose risk reduction strategies, including using less of the tested substance, changing their route of administration to a route with lower overdose risk (e.g., from injecting to snorting), using with others, using more slowly, or discarding their substances completely (5, 9, 11, 12).

Nascent rural research suggests low awareness of FTS (13, 14). Walters et al. (2023) found about half of the PWUD participants in rural Illinois had previously heard of FTS, and under a quarter reported that they had used FTS to detect fentanyl in their substances. A qualitative study by Allen et al (2020) explored the *hypothetical* possibility of using FTS among rural Appalachian residents who inject drugs, and found that they expressed high willingness to use FTS if ever offered them. Participants further reported that if they were to use FTS and found that their drugs were positive, they might be more likely to use less, do tester shots, or discard the substances.

Expanding this rural research is essential. Barriers to FTS use may vary across urban and rural areas. In particular, the discovery or use of FTS may reveal PWUD status and open PWUD up to stigma (10, 14–16). Stigma may be a greater concern in rural areas in part due to smaller social networks and intensified confidentiality concerns (17–22). Moreover, naloxone availability is lower in rural than in urban and suburban regions (23, 24), and so efforts to prevent overdoses before they start might be paramount.

The present qualitative analysis thus explores rural Appalachian PWUD’s perspectives on an FTS distribution and education intervention. We were guided by Rhodes’ Risk Environment Framework (REF), which supports analyses of how different dimensions of environments (physical, social, economic, healthcare, policy) and levels of influence (micro, meso, macro) intersect and influence overdoses and other drug-related health outcomes (25, 26). The analysis is designed to explore (1) participants’ perceptions of risk environment features that shape their interest in and use of FTS; (2) their experience of the FTS intervention itself and their use of FTS; and (3) their responses to positive test results. Based on emerging findings, we also explore participants’ dissemination of the intervention.

## Methods

### Intervention description

All participants in this qualitative analysis took part in the CARE2HOPE (C2H) parent study, which assessed the extent to which PWUD benefitted from a healthcare navigation, HIV/HCV testing, and overdose education and prevention intervention. C2H encompassed 12 rural counties in the heart of Appalachian Kentucky’s opioid crisis; six of these counties were randomized to the intervention condition. Intervention targets and components and the target population were selected by eight community-academic partnership groups (CAPs) that spanned these 12 counties (some CAPs covered two small counties). The CAPs developed the intervention so that it focused on PWUD who were involved in the criminal-legal system (CLS). The intervention’s primary targets included reductions in the frequency of illegalized drug use; a secondary target was reduced overdose rates. Intervention components selected by the CAPs included FTS and naloxone distribution and education; motivational interviewing and health navigation to reduce drug-related harms; and HIV and HCV counseling, testing, and linkage to care. C2H intervention sessions and healthcare navigation services were delivered by project staff called “Rural Health Navigators” or “REHNs” who were residents of the communities they served; most reported that they were in recovery. As a part of the first intervention session, REHNs offered FTS (roughly 10 per session), showed a video about how to use FTS, and answered participants’ questions about FTS use; approximately 10 additional strips were offered in monthly in-person session for the next three months, along with booster educational sessions as needed. The video was created by St. Ann’s Corner of Harm Reduction, and covered the following topics: overdose statistics; fentanyl potency; protocols for using FTS to test substances in pill, powder, or liquid formulations; how to interpret test results; possible harm reduction strategies when results were positive; and limitations of the strips (e.g., may not identify all varieties of fentanyl). The video lasted 4 minutes and 45 seconds, and is freely available (https://www.youtube.com/watch?v=gIovAAV-Amg).

### Sample and recruitment

Individuals were eligible for the C2H intervention if they lived in one of the six Kentucky counties randomized to the intervention condition; were 18 years old or older; had been engaged in the CLS in the past 30 days prior to screening; and either used opioids to get high or injected drugs to get high 30 days prior to the screening or to CLS involvement. CLS involvement was defined broadly to include arrest; incarceration; community supervision (probation or parole); or involvement with the courts, including drug and family court; and the Child Protective Services system. Individuals were recruited into C2H via multiple community-based pathways, including tabling outside probation and parole offices, courthouses, and harm reduction programs; holding cookouts near places where eligible individuals might live or seek services; flyering; and snowball sampling.

To be eligible for this qualitative sub-study, individuals had to have been enrolled in the C2H intervention for at least three months. The team purposively sampled qualitative participants from the C2H cohort, seeking to create a sample of participants who varied by county and gender.

### Data Collection

In-depth, semi-structured interviews were conducted between March and October 2022 ($30 honorarium). Study staff conducted interviews over a HIPAA-protected Zoom interface; interviews lasted between 30–100 minutes. Participants without access to private space and/or Zoom-equipped technology utilized C2H office space; REHNs were not in the offices during interviews.

The interview guide was informed by literature, REF, and C2H REHNs. The guide covered participants’ social environments (e.g., family support), economic environments (e.g., financial needs, employment barriers), healthcare engagement, CLS engagement, and physical environments. One interview domain covered participants’ experiences with FTS, including receiving intervention-provided education, and utilizing FTS; in response to findings from early interviews, we added questions about sharing and discussing FTS with participants’ social networks.

### Analysis

Interviews were transcribed, audio-recorded, and anonymized. Analyses were then undertaken using NVIVO software. The team developed and iterated the codebook using inductive and deductive coding approaches. Deductive codes were derived from the interview guide, REF domains, and literature, while inductive codes emerged through interview memos, discussions and thematic patterns.

Data were analyzed using a constructivist thematic approach in which we immersed ourselves in the transcripts through multiple readings; used compare and contrast methods and memos to identify salient patterns (“themes”) threading across transcripts; labelled and defined these themes; and sought negative cases. We drew on REF during pattern identification, labeling, and definition steps, exploring the salience of different REF dimensions and levels.

## Results

Twenty-nine people took part in the qualitative interviews. Consistent with the sociodemographic composition of the area, almost all were White (Table 1). Most participants were women (62%) and the sample’s average age was 34 (range 19–58). Approximately one third had not graduated from high school or earned a GED, and another third reported that their highest educational attainment was a high school degree or GED. The most commonly reported substances of choice were methamphetamine (41%) and heroin (38%).

Major themes identified were (1) pervasiveness of fentanyl and fentanyl overdoses in this rural risk environment; (2) need for primary prevention tools to avoid fentanyl overdose in this rural risk environment; (3) experiences of the FTS intervention and FTS use; and (4) participants’ dissemination of the FTS intervention throughout their networks. Data from all 29 participants were analyzed to generate Theme 1 (pervasiveness of overdoses), Theme 2 (need for primary prevention tools), and Theme 4 (FTS dissemination). At the time of the intervention, 10 participants had suspended/ceased use (drug-related eligibility criterion pertained to the 30 days before incarceration or before the start of probation/parole, for those individuals engaged in those dimensions of the CLS). These participants did not contribute data to analyses of FTS use in Theme 3.

### Theme 1: Pervasiveness of fentanyl and fentanyl overdoses

Participants – regardless of whether they were in active use during the three-month intervention – reported that fentanyl was present in much of the unregulated drug supply, elevating overdose risk for local PWUD. Fentanyl saturated the local drug market, particularly supplies of methamphetamine, heroin, and Xanax. This saturation generated a surge in overdoses in the region:

[There has been] a huge increase in heroin/fentanyl in the area and it’s just absolutely wiping folks out. Like it is just dropping people like flies.

31-year-old man

This surge was felt acutely in these small rural communities “because it’s a small little town and everywhere you go around the corner somebody’s OD’d.” The loss of friends and family in these “small little towns” was devastating:

I’ve had so many friends die, that didn’t make it through, man, and it bothers me. I think about them all the time.

34-year-old man

In addition, many participants reported personal experiences of near-fatal overdose. As one 34-year-old woman remarked, “I’ve overdosed seven times and flatlined, so I’m lucky to be alive…” Some reflected on the cognitive dissonance that such a small amount of a single substance could cause so much devastation to themselves and their community,

“I don’t understand it…Such small a thing, such a big quantity of people [dead].”

34-year-old man

### Theme 2: The need for primary prevention tools to avoid fentanyl overdose in this rural risk environment

Interviews with participants – regardless of whether they were in active use at the time of the intervention – highlighted the need for primary prevention tools to avoid fentanyl overdoses in a rural risk environment where lay-administered naloxone was both necessary and insufficient, and where Emergency Medical Systems (EMS) were unreliable and often undesirable.

Participants reported multiple instances in which they and others had used lay-administered naloxone to revive overdosing friends, family members, and strangers; we see this as an important PWUD-led feature of the local healthcare environment. As one participant recounted,

Without people [having naloxone], I’d say that there would’ve been twice, if not triple the amount of overdoses, so I really think that it saved a lot of people’s lives.

36-year-old woman

While lay-administered naloxone was absolutely necessary in this setting, participants keenly felt its limitations. Each overdose reversal took a physical toll on survivors. When asked what it felt like to be “hit” with naloxone, one participant explained:

It felt like my whole body getting run over by train…it saves your life [but]…every muscle in your body tightens up. You freeze to death, you puke. It’s awful.

36-year-old woman

Reversals also took an emotional toll on PWUD who survived and on their family members, friends, and the community, as illustrated by the commingling of gratitude with grief in this recounted experience:

I gave [my neighbor] some Narcan. It was her husband [who later] OD’ed inside the car. She used that Narcan, he comes out of it… [After he was released from the hospital] I go to step outside my door to leave and…[the husband] comes down, he hugs me, starts crying and stuff…He said, “If it wasn’t for you, I would’ve been gone.” And [his wife] come down the hill. She was crying…

36-year-old man

Moreover, intersecting features of the physical and CLS risk environments in this rural area made EMS an unreliable and sometimes undesirable partner in overdose reversals. Individuals often used substances in unmapped areas like national and state parks where EMS was unable to find them:

[Someone overdosed and] I said, “Well, I will call 911.” So I called them. They said they couldn’t send out anybody because I didn’t know where to send them to. I knew where I was at, but I didn’t know house number. So they said they couldn’t send anyone.

46-year-old woman

Likewise, long travel distances in these hilly rural areas could also render EMS moot: PWUD experiencing an overdose found themselves suffering through long wait times for EMS to arrive, if they arrive at all. As one participant summarized:

In rural areas, the ambulance doesn’t always come… time is very precious in those moments. Every second counts.

30-year-old woman

Moreover, participants reported concerns that the police would accompany EMS, and thus that anyone present could be arrested because of drugs or paraphernalia at the scene, open warrants, or probation/parole status, or be exposed to degrading encounters with law enforcement. As one participant remarked,

[Calling 9-1-1] makes me nervous because I know the law’s coming with it…[I had to call 911 before] and I didn’t like the way they treated [the person who overdosed]… Cause it kind of like [the law enforcement officers were] messing [with him] because [the guy] had accidentally overdosed or whatever, but that was the first time he had ever done that. [The police] treated him like he was nothing.

30-year-old woman

Collectively, these findings suggest that primary prevention tools to stop overdoses from occurring at all were crucial in this rural risk environment.

### Theme 3: Experiences of the C2H FTS intervention and FTS use

Participants – regardless of whether they were in active use at the time of the intervention – reported that FTS were scarce in the community prior to the C2H intervention. Most participants (n = 22 of 29, 76%) had never heard of FTS before taking part in the intervention. As a result, participants reported that the video-based FTS training was informative:

It taught me a lot. It taught me how to use the strips, taught me how to check the dope or the pills or whatever I’ve got a baggie of. How to dilute the water and how to do the test strips correctly. So it was a good little experience.

36 year-old woman

As noted, 10 participants were not using at the time of the intervention. Of these 10, seven accepted FTS strips but did not use them personally (see Theme 4 for some discussion of these and other participants). The remaining 19 participants accepted the strips, with the exception of one who reported accessing them elsewhere. Of these 19 participants, 11 recalled a positive test result. The remainder of this theme’s description pertains to these 11 participants.

When telling the story of a memorable time involving a positive result, these 11 participants reported deploying one or more of the following strategies: (1) using less of the substance (n = 10); (2) throwing it away (n = 6); and (3) using the substance with no change (n = 4). We describe each response in turn.

Most participants (n = 10) explained that a positive FTS result changed *how* they used the substance, though it did not stop them from using it:

Yeah, they didn’t keep me from using the dope…but it definitely had me more cautious of how much I was using. Yeah…If I used the strip and it tests positive for fentanyl, then I’m going to do about half of what I was about to do.

31-year-old man

As another stated,

You can always do more [drugs], but you can’t take away… I was more careful.

26-year-old woman

In contrast several participants (n = 6) used the FTS to avoid fentanyl-laced substances altogether:

I won’t do fentanyl or anything like that. And I deal with nobody that does because I don’t want to deal with the overdose part. I don’t want that.

46-year-old woman

Finally, four reported that a positive test result did not alter how they used the substance. These participants expressed that they had a high tolerance or that they were “a fentanyl junkie” who sought out fentanyl or substances mixed with fentanyl. One participant explained:

[The positive FTS result] made me be like, “Oh, I’m definitely going to do all of this…because my tolerance is high”.

32-year-old man

Regardless of whether a positive test result altered their use pattern, participants reported that FTS were an easy to use, effective tool that informed their substance use experiences and practices in an unregulated drug market. Overwhelmingly, FTS were described as “lifesaving”:

At first, when I first heard about it, I didn’t really think nothing about it, but it could be really lifesaving…. Especially if you don’t know [if fentanyl] is in [your drugs]…It’s wild that this program put [FTS] out there because we needed it. We really did.

46-year-old woman

For many participants, FTS assuaged some of their apprehension about using substances in an unregulated market:

I wasn’t scared of the dope I had anymore because I could test it myself.

36-year-old woman

### Theme 4: Participant FTS dissemination

Although not the intention of the intervention, 10 of the 29 participants (34%) shared FTS with friends, family, or other PWUD members of the community. These 10 participants included 7 who were not in active use at the time of the intervention.

I gave a couple [of FTS] to my brother [and] my boyfriend’s cousin…I gave a couple to my sister…They’d use heroin and maybe didn’t know that fentanyl would be in there…and I’d be like, “Well here, if you’re doing it, test it before you do it and that way, you know.”

36 year-old woman

One participant discussed sharing FTS with a local person who sells drugs so they could proactively monitor the supply they were selling:

The person who was selling said, “I never thought about doing that. Never thought about testing them, nothing.” I’m like, “Well, here you go. Here’s you some strips. Test them when they come in”…There’s been a couple times they’ve tried to slip [fentanyl] in on him and he’s got rid of them. He’s lost money. He’d rather lose money than kill somebody.

36-year-old man

Overall, participant discussions of FTS exemplified a strong desire to protect their community from overdose and more trauma:

I hear them talking about that there could be fentanyl [in what they’d bought]…Cause they thought they was doing heroin, I guess. And I said, “here is [a strip so] maybe you can test and see if it’s got fentanyl in it. So maybe you don’t do so much of it and not kill yourselves.”

30-year-old woman

## Discussion

In this analysis, rural Appalachian PWUD experienced FTS as a lifesaving intervention that helped them survive an especially hazardous risk environment. As elsewhere (6, 14), fentanyl saturated the local drug market. Few, however, of these rural participants had heard of FTS before participating in the intervention. As found in cities, almost all PWUD who were actively using during the intervention welcomed the strips when offered them, and positive test results generated changes in use patterns designed to minimize overdose risk (7, 9, 12, 27–32). Particular features of this rural Appalachian risk environment – specifically, unreliable EMS response, fear of accompanying law enforcement, and concerns about the physical and emotional toll of naloxone reversals – sharpened interest in FTS as a primary prevention method that stopped overdoses from occurring at all. Recognizing PWUD responses to this intervention is especially important in light of current federal attempts to dismantle evidencebased harm reduction interventions in the US (33, 34).

Specific interplays of rural risk environment features rendered FTS a particularly essential tool for these PWUD. PWUD remarked that EMS was unreliable. PWUD often used substances in parks and other unmapped areas, and so bystanders were not always able to report an address to 911 dispatchers. Moreover, long travel distances – over Appalachian hills, around pastures, crossing rare bridges – often meant that ambulances could not arrive in time. PWUD also discussed the specter of arrest or degrading treatment if law enforcement accompanied EMS. While this last concern has been broadly reported, we note that this feature of the policy environment is especially active in these rural Appalachian counties, where county-level incarceration rates were far higher than incarceration rates in the 12 most populous US counties in the years preceding data collection (35). These local intersections of features of the healthcare, physical, and policy environments rendered participant-led harm reduction solutions paramount for PWUD. In addition to these local nuances participants also reported that, though naloxone was an essential tool to reverse overdoses, reversals were physically painful and could be emotionally devastating for bystanders and survivors alike. FTS offered a PWUD-controlled, prevention-focused method to prevent an overdose from occurring in the first place.

Notably, while the C2H FTS education and distribution intervention was designed to operate within the microlevel healthcare environment domain, participants transformed it into a mesolevel healthcare intervention by leveraging their social networks, an essential component of the Appalachian social environment. Participants reported sharing FTS strips and test results with their partners, friends, and family members; one sought to persuade a local person who sells drugs to test all the substances they sold. Other studies have similarly reported that PWUD are willing to distribute FTS to others who may benefit (30, 36–38). In this particular setting, social networks are a potent dimension of the local social environment: social and drug co-usage networks of PWUD in this Appalachian rural area are densely connected (39). Future interventions could intentionally leverage this resilient feature of the local social environment, and support PWUD efforts to diffuse FTS knowledge and strips via their social networks. There is a long history of analyzing PWUD networks in this region (39) and this could be extended to quantify the spread of FTS-related social norms, knowledge, and self-efficacy.

Findings should be interpreted in light of limitations, which we present using Maxwell’s validity framework (40). Descriptive validity (i.e., the extent to which we captured what was said) was strengthened by using verbatim transcripts and comparing the transcripts to audio recordings. Interpretive validity (i.e., the extent to which the researcher captured participants’ meanings) was enhanced through extensive reflective and descriptive memo writing. Transcripts were coded by two researchers (MF and ZK) and reviewed by HC. In depth discussions were held to minimize bias around codes, and code applications. Any disagreements or discrepancies were discussed and resolved among the analytic team.

## Conclusion

Results suggest that FTS education and distribution are essential tools to help reduce overdose occurrence and mortality in this rural risk environment, where EMS response can be unreliable and may be accompanied by degrading law enforcement treatment; it also revealed that naloxone was essential but inadequate, because of reversals’ physical and emotional toll. Future studies should intentionally leverage networks – a key strength of this region’s risk environment – to diffuse FTS interventions.

## Figures and Tables

**Table 1:** Description of Qualitative Sample (N = 29)

Characteristics	Frequency	Percentage
**Sex**		
*Male*	11	37.9
*Female*	18	62.1
**Age (mean, range)**	34 (19–58)	
**Educational attainment**		
*Less than high school*	10	34.5
*High school diploma or GED*	10	34.5
*Some college*	8	27.6
*Associates degree/trade or technical school*	1	3.5
**Race**		
*White*	28	96.6
*Other*	1	3.5
**Current drug of choice for getting high**		
*Methamphetamine*	12	41.4
*Heroin*	11	37.9
*Opioid painkillers*	2	6.9
*Buprenorphine*	2	6.9
*Benzodiazepines*	1	3.5
*Gabapentin*	1	3.5

## Data Availability

Because of the illegalized nature of the topic, the potential identifiability of participants in these small rural counties, and the impossibility of anonymizing participants’ stories, we have not deposited data in a publicly accessible archive. Data are available upon request.
